# A Simple Technique for the Positioning of a Patient with an above Knee Amputation for an Ipsilateral Extracapsular Hip Fracture Fixation

**DOI:** 10.1155/2013/875656

**Published:** 2013-12-12

**Authors:** N. Davarinos, P. Ellanti, G. McCoy

**Affiliations:** Department of Trauma Orthopaedics, Waterford Regional Hospital, Dunmore Road, Waterford, Ireland

## Abstract

The positioning of the patient on the fracture table is critical to the successful reduction and operative fixation of hip fractures which are fixed using the dynamic hip screw system (DHS). There is a standard setup which is commonly used with relative ease. Yet the positioning of patients with amputations either above or below knee of the affected side can pose a significant challenge. We describe a novel positioning technique used on a 51-year old patient with a right above knee amputation who sustained an intertrochanteric extracapsular hip fracture.

## 1. Introduction

Hip fractures are a common source of morbidity and mortality worldwide [1]. The incidence of hip fractures is increasing and is projected to triple by 2050 [2]. Dynamic Hip Screw (DHS) is a well-established implant in the fixation of extra-capsular hip fractures [3]. The positioning of the patient on the fracture table is critical to the successful reduction and operative fixation of the fracture. This generally involves the unaffected side being flexed at the hip and knee and positioned to allow the fluoroscopy machine access to the affected side. The foot of the affected side is positioned in a boot attached to the reduction table through which traction and rotation as being appropriate can be applied to reduce the fracture and maintain an acceptable position during the surgical fixation.

This standard setup is quick and is achievable with relative ease in patients with intact lower limbs. The positioning of patients with amputations either above or below knee of the affected side can pose a significant challenge. We describe a positioning technique used on a 51-year old patient with a right above knee amputation who sustained an intertrochanteric extr-capsular hip fracture.

## 2. Case Report

A 51-year-old male with a right above knee amputation presented to the emergency department with a painful right hip subsequent to a fall on that side. He had been mobilising with two crutches for the previous four weeks while awaiting new prosthesis for the right above knee amputation from previous infected total knee arthroplasty.

**Figure 1:**
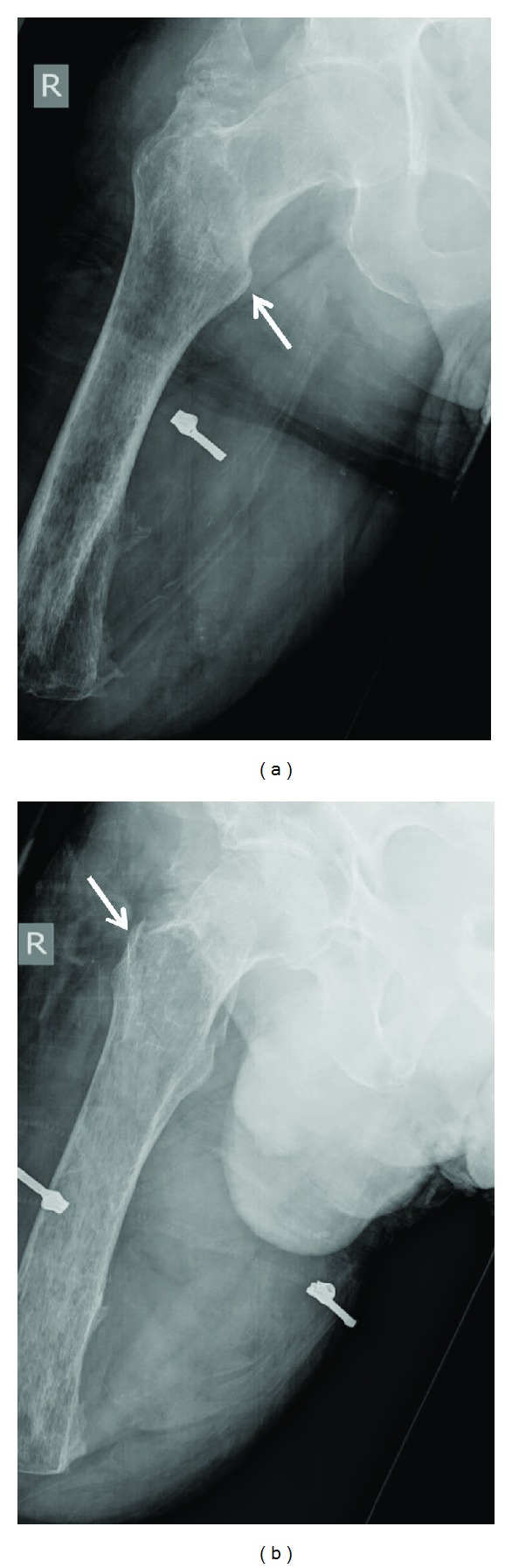
Anteroposterior and lateral radiographs of the right hip demonstrating an undisplaced extracapsular intertrochanteric fracture (white arrow).

Plain film radiographs revealed an undisplaced extracapsular intertrochanteric fracture of the right hip (Figure 1).

**Figure 2:**
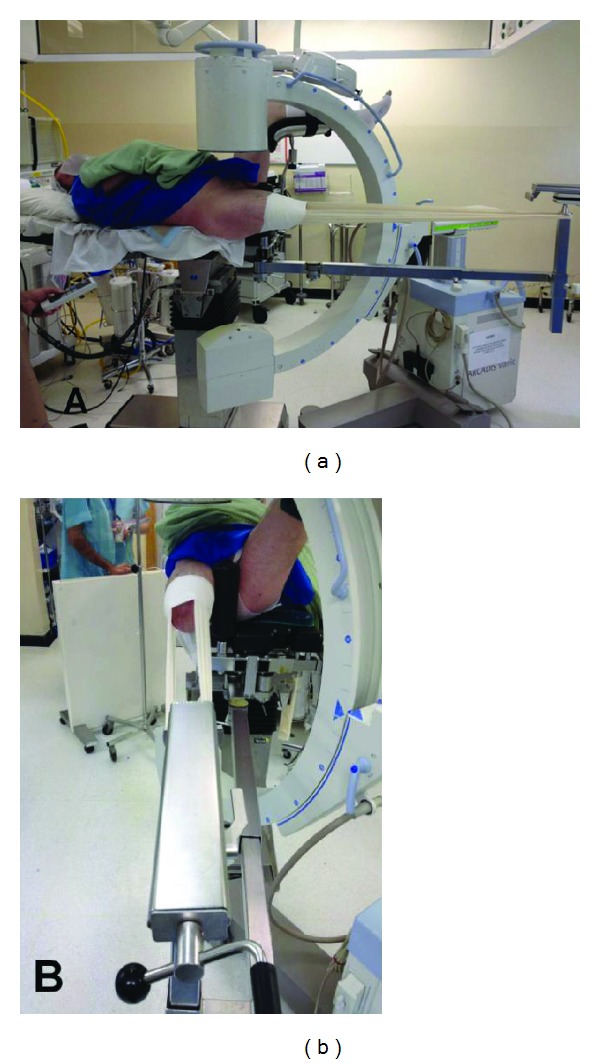
Intraoperative photographs demonstrating the setup of the patient on the fracture table with the amputated limb secured with adhesive fabric tape.

As an active and mobile amputee with a functional prosthesis, the patient was scheduled for a DHS fixation of the right intertrochanteric hip fracture with spinal anaesthesia. Securing the amputated limb poses a challenge in patients with above knee amputations. Our setup involved the standard fracture table used routinely to position a patient for DHS fixation. Adhesive fabric tape (Elastoplast 7.5 cm × 4.5 m by BSN medical UK) was used to secure the stump to the distal end of the fracture table in a similar manner to the application of skin traction. Combining a specially formulated porous adhesive and a superior woven cloth, Elastoplast now Tensoplast, provided satisfactory adhesion while remaining secure in place. Such adhesive fabric tape and a crepe bandage was used circumferentially around the distal stump to further secure the limb (Figure 2).

**Figure 3:**
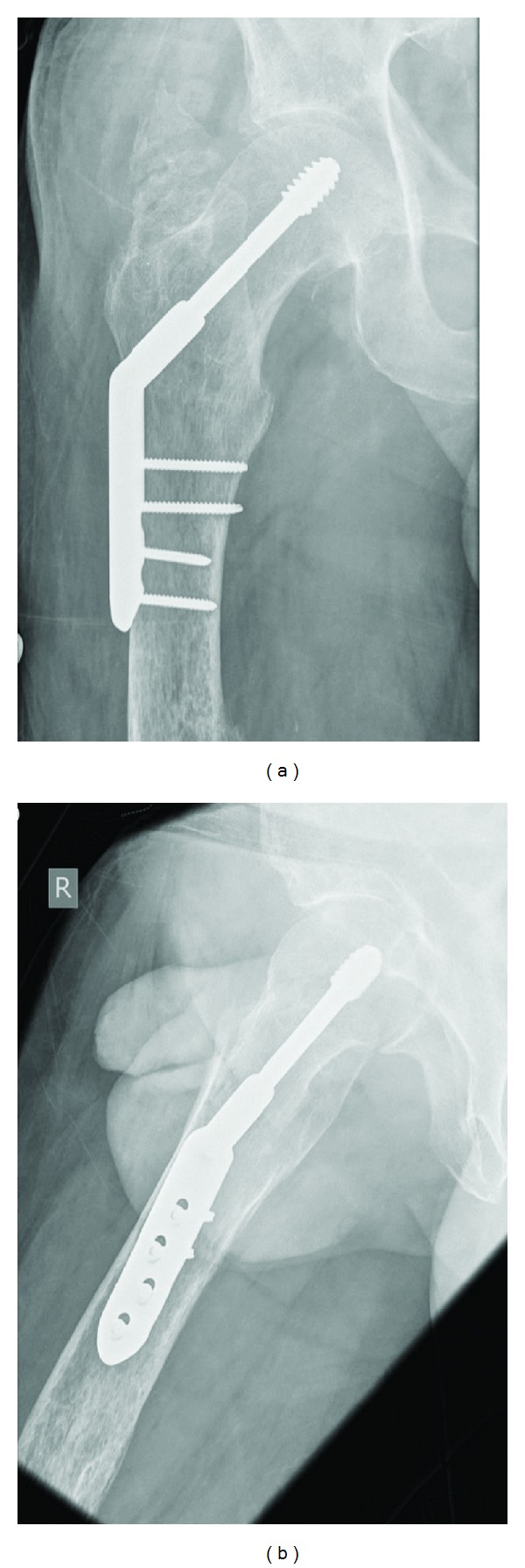
Postoperative radiographs demonstrating satisfactory DHS fixation of the right intertrochanteric hip fracture.

The DHS fixation was uncomplicated and the patient's recovery was uneventful. The patient was mobilised full weight bearing as tolerated with physiotherapy and discharged two days postoperatively. The post operative radiographs were satisfactory (Figure 3) and at the final follow up at three months, the patient reported a full return to pre-injury functional status.

## 3. Discussion

There is little in the literature regarding the management of intertrochanteric hip fractures in patients with ipsilateral above or below knee amputations. Securing the amputated limb to the fracture table poses a significant challenge. Al-Harthy et al. [4] described inverting the boot to fit around the knee and stump in a below knee amputee to facilitate positioning the application of traction if necessary. Rethnam et al. [5] described positioning of the below knee amputated limb on a radiolucent leg support. Their patient had an undisplaced fracture that did not require traction and rotation control was provided by an assistant.

Limbs that have undergone an above knee amputation can have short stumps that can be very challenging to position or apply traction to. To our knowledge, there is only one report on the positioning technique for patients with a fracture and an above knee amputation. Aqil et al. [6] described resting of the stump on a radiolucent leg support during operative fixation similar to Rethnam et al. Their patient was a bilateral above knee amputee with a minimally displaced intertrochanteric hip fracture.

Our technique is an alternative to those described above. While the skin traction facilitated positioning of the limb, it provided little rotation control. Our patient had an undisplaced fracture and our technique resulted in a timely and satisfactory fixation. Due to the size of the stump and the small area the skin traction is applied to, it cannot generate any significant amount of traction prior to it getting unravelled. It may be conceivably more useful in below knee amputees where adequate skin traction and therefore traction may be applied. The skin around the stump needs to be carefully inspected prior to application of this technique as any fragile areas may become prone to breaking down during the procedure if prolonged.

Some level of rotation can be applied using a wooden stick or a metal bar placed within the crepe bandage limbs and applying rotation towards the desired direction. Yet if significant traction and rotation control were needed for the reduction and fixation of these fractures, the use of skeletal pin traction may be necessary. The application of such a pin carries with it the risk of injury to the soft tissues of the stump. The pin may pull out of the osteoporotic bone on application of traction.

## 4. Conclusion

There is little in the literature regarding the intraoperative positioning of patients with a hip fracture and an above knee amputation. There is one previous report and we describe an alternative technique for the positioning of a patient with an extracapsular hip fracture and an ipsilateral above knee amputation.
